# Antipsychotic-Like Effect of the Muscarinic Acetylcholine Receptor Agonist BuTAC in Non-Human Primates

**DOI:** 10.1371/journal.pone.0122722

**Published:** 2015-04-16

**Authors:** Maibritt B. Andersen, Carrie Hughes Croy, Ditte Dencker, Thomas Werge, Frank P. Bymaster, Christian C. Felder, Anders Fink-Jensen

**Affiliations:** 1 Research Institute of Biological Psychiatry, Sct. Hans Hospital, Roskilde, Denmark; 2 Lilly Research Laboratories, Eli Lilly and Company, Indianapolis, Indiana, United States of America; 3 Laboratory of Neuropsychiatry, Department of Neuroscience and Pharmacology, University of Copenhagen, Copenhagen, Denmark; 4 Psychiatric Centre Copenhagen, University of Copenhagen, Copenhagen, Denmark; 5 Euthymics Bioscience Inc, 43 Thorndike St Suite 1-3, Cambridge, Massachusetts, United States of America; University of Montreal, CANADA

## Abstract

Cholinergic, muscarinic receptor agonists exhibit functional dopamine antagonism and muscarinic receptors have been suggested as possible future targets for the treatment of schizophrenia and drug abuse. The muscarinic ligand (5R,6R)-6-(3-butylthio-1,2,5-thiadiazol-4-yl)-1-azabicyclo[3.2.1]octane (BuTAC) exhibits high affinity for muscarinic receptors with no or substantially less affinity for a large number of other receptors and binding sites, including the dopamine receptors and the dopamine transporter. In the present study, we wanted to examine the possible antipsychotic-like effects of BuTAC in primates. To this end, we investigated the effects of BuTAC on d-amphetamine-induced behaviour in antipsychotic-naive *Cebus paella* monkeys. Possible adverse events of BuTAC, were evaluated in the same monkeys as well as in monkeys sensitized to antipsychotic-induced extrapyramidal side effects. The present data suggests that, the muscarinic receptor ligand BuTAC exhibits antipsychotic-like behaviour in primates. The behavioural data of BuTAC as well as the new biochemical data further substantiate the rationale for the use of muscarinic M_1_/M_2_/M_4_-preferring receptor agonists as novel pharmacological tools in the treatment of schizophrenia.

## Introduction

Cholinergic agents exhibit antipsychotic effects in schizophrenic patients. Pfeiffer and Jenney administered the partial muscarinic receptor agonist arecoline by subcutaneous injection to twenty-three schizophrenic patients and clinical improvement described as “lucid intervals” were reported in more than 80% of the patients [1]. Also preclinical data indicate antipsychotic potential of cholinergic compounds [1,2] even though parasympathomimetic side effects have hampered firm conclusions.

We have earlier reported that the muscarinic ligand xanomeline, historically described as a partial receptor agonist at muscarinic M_1_/M_4_ receptors and antagonist at muscarinic M_2_/M_3_/M_5_ receptors inhibits dopaminergic effects in rodents [3] and *Cebus Apella* monkeys [4]. These data are in accordance with clinical data where xanomeline was reported to reduce psychotic-like behaviour in Alzheimer′s disease patients [5] and psychosis in schizophrenic patients [6].

The muscarinic ligand PTAC, historically described as a partial agonist at muscarinic M_2_/M_4_ receptors and antagonist at muscarinic M_1_/M_3_/M_5_ receptors in cloned cell lines, exhibits functional dopamine antagonism despite its lack of affinity for the dopamine transporter and dopamine receptors [7]. When tested, the pharmacological effects of PTAC could be antagonized by the muscarinic antagonist scopolamine, indicating that the functional dopamine antagonism was mediated through agonist effects at muscarinic receptors [7].

BuTAC, another muscarinic ligand, was earlier reported to inhibit apomorphine-induced climbing [8], apomorphine-induced disruption of pre-pulse inhibition [9] and conditioned avoidance responding [10]. The compound was historically described as a muscarinic M_2_/M_4_ receptor preferring partial agonist with muscarinic M_1_/M_3_/M_5_ receptor antagonistic activity in cloned cell lines [8]. However, a more recent functional in vitro assay provides a direct measure of G protein activation, which allows enhanced signal sensitivity by reducing the background and has proven critical for the Gq-coupled receptors (M_1_, M_3_, M_5_) [11]. In this assay, the muscarinic receptor activity profile of BuTAC was slightly revised [10] i.e. BuTAC exhibits full agonist activity at the M_2_ receptor, partial agonist activity at the M_1_- and M_4_ receptor and full antagonist activity at the M_3_- and M_5_ receptor [10]. Consequently, we found it relevant to retest the above mentioned muscarinic reference compounds xanomeline and PTAC in the same assay, since detailed knowledge about muscarinic subtypes agonist/antagonist properties of these two muscarinic reference compounds is of importance in order to elucidate the involvement of muscarinic receptor subtypes in the antipsychotic-like behaviour of muscarinic ligands.

The primary behavioural objective of the present study was to examine whether the anti-psychotic-like effects of BuTAC observed in rodents is reflected in non-human primates, using an animal model closely mimicking the human biological and behavioural system. To this end, we studied amphetamine-induced behaviour in antipsychotic-naïve *Cebus Apella* monkeys. Sequence analysis performed between human and new world monkey species showed high sequence identity (>90%), with no evidence of sequence variations for residues forming the orthosteric binding pocket, and thus all the in vitro pharmacological evaluation of these ligands shown should be translatable to the behavioural studies performed in non-human primates.

The secondary behavioural objective of the study was to investigate possible side effects of BuTAC by testing the compound both in antipsychotic-naïve monkeys as well as in monkeys sensitized to extrapyramidal side effects (EPS) by earlier long-term treatment with first generation antipsychotics.

## Materials and Methods

### In Vitro Muscarinic Selectivity Profile

The antibody capture scintillation proximity technique previously described by DeLapp et al [11] was used in a 96-well plate to determine GTP-γ-[35S] binding. One hundred μl (20–40 fmol/well) of membrane preparation from CHO cells that stably express the human M_1_-M_5_ muscarinic receptors (PerkinElmer Life and Analytical Sciences, Boston, MA) were incubated with varying concentration of test compound (final concentrations from 50pM to 1μM) for 30 min. One μM final concentration of GDP was added to M_2_ and M_4_ receptor membranes before incubation. The antagonist curves were generated by use of a concentration that gave a response of 90% of maximum effect (EC90) of the full agonist oxotremorine-M. After the incubation period, 50 μl of diluted GTP-γ-[35S] (500 pM final concentration; Perkin Elmer Life and Analytical Sciences) was added to each well followed by 30 min of incubation. Then the labelled membranes were solubilized with 0.27% Nonidet P40 for 30 min where after they were incubated with 20 μl (final dilution of 1:400) of the appropriate rabbit polyclonal Gα antibody (Covance, Princeton, NJ) for 60 min. An anti-Gαq/11 antibody was used for the M_1_-, M_3_-, and M_5_ membranes, whereas an anti-Gαi-3 antibody was used for the M_2_- and M_4_ membranes (Eli Lilly and Company, Indianapolis, IN). After 60 min of incubation, 50 μl (1.25 mg/well) anti-rabbit scintillation proximity assay (SPA) beads (GE Healthcare, Piscataway, NJ) were added per well where after the plates were incubated for another 3 h. By use of a Wallac MicroBeta TriLux scintillation counter (PerkinElmer), the plates were centrifuged for 10 min at 700 × g and counted for 1 min per well. The incubations took place at room temperature in GTP-binding assay buffer (20 mM Hepes, 100 mm NaCl, 5 mM MgC12, pH 7.4). The data were analyzed by use of nonlinear regression for a sigmoidal concentration response curve (GraphPad Prism v. 4.0). Data were normalized to a full agonist response (100 μM oxotremorine-M). Thereafter, the percent efficacy (%E_max_) was expressed as a percentage of maximum 100 μM oxotremorine-M signal. The antagonist percent inhibition data were normalized to data generated with an EC90 concentration of acetylcholine. The mean relative EC_50_, IC_50_, and %E_max_, and pKi values were calculated as a mean of at least three independent experiments±SEM. In order to obtain relevant comparisons between subtypes, the experimental system is routinely calibrated with reference agonists (oxotremorine-M, pilocarpine, MCN-A343) to measure full and partial agonism as well as to ensure that the system is not under- or over-reporting functional efficacies. The radioligand binding assays have earlier been described [12].

### Primates

Seven male Cebus Apella monkeys were used for evaluation the anti-amphetamine effect and the side effect profile of BuTAC. These monkeys were never before administered antipsychotics and had never experienced EPS, but had previously been administered amphetamine. For further evaluation of the EPS potential of BuTAC, the compound was tested in four male and female monkeys, all sensitized to drug-induced EPS by earlier chronic treatment with classical antipsychotic drugs [13].

The EPS sensitized monkeys were originally caught in the wild and had been at the laboratory for 13–20 years at the time of testing. The EPS naïve monkeys were born in captivity and had been at the laboratory for 7 years at the time of testing. They were between 11 and 18 years old, when the studies were initiated. The monkeys weighed between 2.5 kg (smallest female) and 5 kg (largest male). Their weight was monitored weekly and they were regularly checked by a veterinarian.

The two groups of monkeys were housed in separate cages in two temperature- and humidity rooms at a 12-hour light/dark cycle. The monkeys had visual and auditory contact to each other within the room. Once a week they were given freshly cut branches and twigs to chew and play with, and they had a variety of toys available at all times except during experimental sessions. There were radio and television in the animal house and these were turned on during the daytime except during experimental sessions. The monkeys were fed food pellets and a variety of fruits and vegetables. The small females (≤3kg) were kept in cages of 0.70*0.63m^2^ * height 0.85m. The bigger males (4–5 kg) were kept in cages of 0.83*0.76m^2^ * height 1.3m.

All monkeys received all treatments with at least one week between tests and thus served as their own control. To avoid extended physical contact with the experimenter, the cages were reduced in sized by moving the back wall of the cage closer to the front wall to lightly immobilize the monkey while injecting the test substance in the thigh. This was done at 9 am at test days. After the appropriate pre-treatment time, the monkeys were videotaped in their cages at predefined time points after drug injection.

The experimental procedures carried out in this study were in accordance with the European Communities Council Directive of the 24th of November 1986 (86/609/EEC) and had been approved by the Danish Committee for Animal Research.

### Compounds

BuTAC ((5R,6R) 6-(3-Butylthio-1,2,5-thiadia-zol-4-yl)-1-azabicyclo-[3.2.1]-octane), PTAC (5R,6R) 6-(3-propylthio-1,2,5-thiadiazol-4-yl)-1-azabicyclo-[3.2.1]-octane) and xanomeline were synthesized at Eli Lilly and Company. For the behavioural experiment, BuTAC was dissolved in peanut oil. *d*-Amphetamine sulphate (*d*-Amphetamine) was purchased from Sigma Chemicals (St. Louis, MO) and dissolved in 0.9% saline.

### Behavioural Testing Procedures in Primates

The rated primate behaviours and symptoms were the following: unrest, stereotypy, arousal, sedation and bradykinesia (Table 1). All monkeys were videotaped in 90-sec. sessions at specific time points throughout the test sessions. The videotapes were rated for amphetamine-induced behaviours and EPS by two experienced raters blinded to the treatment of the animal by means of a rating scale adapted from Peacock et al. [13,14] and previously described by Andersen et al. [4] (Table 1). In case of any discrepancy between the two ratings, the recording in question was re-evaluated and a rating agreed upon. The monkeys were videotaped at t = 30, 60, 90, 135, 150, 165, 180, 240, 300 min after BuTAC (or vehicle) administration. For the combination studies, d-amphetamine was injected at t = 120 min. Oral dyskinesia was measured in counts per 90 sec. The severity of other behaviours and symptoms were rated on a scale ranging from 0 (not present) to 6 (extreme presence).

**Table 1:** Description of behaviours and rating scales.

Behaviour	Description of behaviours and rating scales	Scale
Unrest	Restlessness including fidgeting and frequent changes of direction of movement or frequent changes between different behaviours.	0–6
Stereotypies	Repeated futile movements, abrupt whole body movements and aborted behaviours.	0–6
Arousal	Degree of vigilance ranging from not awake to extreme vigilance in relation to self or the environment.	0–6
Sedation	Degree of drowsiness ranging from fully awake to heavy sleeping (cannot be awakened by gross stimuli).	0–6
Oral dyskinesia	Jaw movements and tongue protrusions.	Counts/90 s.
Bradykinesia	Slow and/or stiffened movements ranging from normal tempo and flexibility to fixed maintained postures.	0–6
Dystonia	Clonic movement of head, neck, limbs and trunk. Gaping and grimacing.	0–6

The scores were based on the following definitions 0, not present; 1, extremely mild (can be a variation of the normal); 2, mild (slightly more pronounced than normal); 3, mild to moderate; 4, moderate to severe (behaviour pronounced but discontinuous); 5, severe; 6, extremely severe (behaviour continuous).

For evaluation of anti-amphetamine effects, BuTAC was tested in doses of 0.001, 0.003, 0.01 mg/kg in combination with 0.5 mg/kg of d-amphetamine. BuTAC or peanut oil vehicle was injected s.c. at approximately 9 a.m. Pre-treatment time before s.c. injection of amphetamine was 120 min. For evaluation of side effects, BuTAC was tested alone at doses of 0.001, 0.003 and 0.01 mg/kg in antipsychotic-naïve monkeys and at doses of 0.003 and 0.01 mg/kg in monkeys sensitized to extrapyramidal side effects (EPS) by earlier long-term treatment with first generation antipsychotics. On the test days, the monkeys did not have access to food or water prior to or during the experiment. None of the animals were sacrificed.

### Data Analysis

Data from the experiments where BuTAC was administered alone collected at t = 60, 180, 300 min are presented. For the d-amphetamine / BuTAC study, data collected at t(amp) = 60, 120 and 180 min (t = 180, 240, 300 min) are reported. Data were analyzed for overall treatment effects at each time point by means of the non-parametric Friedman’s test for repeated measures. The Student Newman-Keuls comparison procedure was used to analyze for specific dose effects. For all tests, the accepted level of significance was p<0.05.

## Results

### Functional Effects of Xanomeline and PTAC at Muscarinic Receptor Subtypes

**Fig 1:**
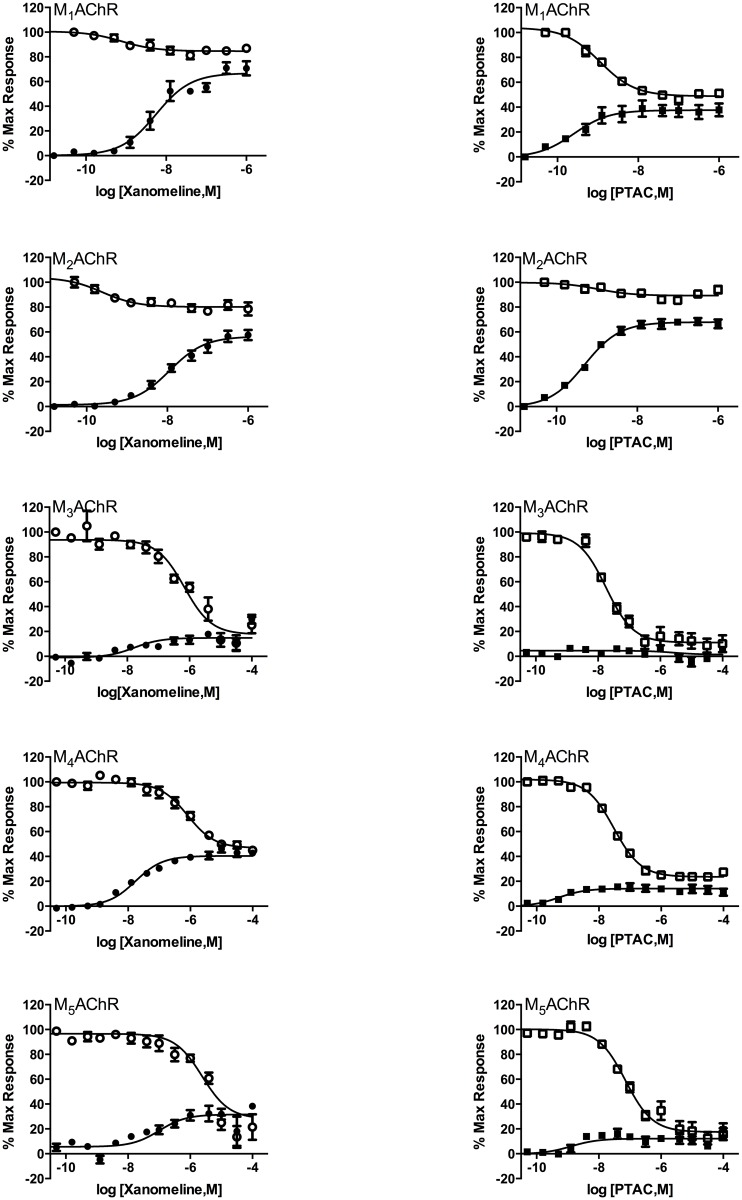
The effect of PTAC and Xanomeline on GTP-γ-[35S] binding in membranes prepared from CHO cells expressing hM_1_-hM_5_ muscarinic receptor subtypes. The agonist (closed symbol) and antagonist responses (open symbols) are expressed as the percentage of maximal signal observed in the presence of the 100 μM ACh. The antagonist data represent the relative antagonism of the agonist response using an experimentally determined EC_90_ concentration of ACh. All data points present the mean ± SEM of at least three separate experiments, each performed in duplicate.

The functional selectivity of the muscarinic agents PTAC and xanomeline, as determined by GTP-γ-[35S] binding, were investigated across the five human muscarinic receptor subtypes (Table 2 and Fig 1). Concentrations in Table 2 are expressed as means and SEMs of the negative log-transformed values (M).

**Table 2:** Competitive binding and GTP-γ-S activity effects of PTAC and xanomeline on the M_1_-M_5_ receptor subtypes.

	M_1_AChR	M_2_AChR	M_3_AChR	M_4_AChR	M_5_AChR
**Xanomeline**					
Rel pEC_50_	8.11 ± 8.41	7.94 ± 8.87	7.75 ± 8.03	7.61 ± 8.47	6.93 ± 8.15
% Emax	68.3 ± 5.31	55.2 ± 5.12	13.6 ± 0.56	41.8 ± 1.99	28.7 ± 0.70
Rel pIC_50_	8.83 ± 9.18	8.78 ± 8.94	6.08 ± 6.77	6.07 ± 6.62	5.48 ± 6.14
% Inhibition	15.2± 0.47	19.4 ± 0.77	87.6 ± 6.79	51.4 ± 2.61	81.8 ± 18.5
pKi	7.03 ± 8.31	6.53 ± 7.44	7.56 ± 8.12	7.48 ± 8.17	7.38 ± 7.91
**PTAC**					
Rel pEC_50_	9.43 ± 9.82	9.30 ± 11.1	8.71 ± 8.95	8.83 ± 10.3	7.81 ± 7.86
% Emax	38.1 ± 9.94	68.5 ± 5.23	11.9 ± 3.07	19.4 ± 0.52	15.4 ± 2.73
Rel pIC_50_	9.03 ± 9.38	8.46 ± 8.55	7.69 ± 8.44	7.61 ± 8.32	6.97 ± 7.33
% Inhibition	54.3 ± 1.70	16.8 ± 5.49	90.45 ± 3.52	92.7 ± 7.49	87.3 ± 7.07
pKi	8.98 ± 9.53	8.42 ± 9.60	9.40 ± 10.8	9.55 ± 11.0	9.43 ± 9.99
**BuTAC**					
Rel pEC50	9.22±9.80	9.56 ±10.26	- ± -	10.27 ± 10.31	7.63 ± 7.41
% Emax	33.56±6.29	136.53 ± 17.20	- ± -	35.53 ± 11.51	10.77 ± 10.87
Rel pIC50	9.03±10.01	- ± -	8.55 ± 8.59	9.19 ± 9.13	8.29 ± 8.16
% Inhibition	57.27±2.35	- ± -	100.00 ± 0.00	57.55 ± 3.22	96.31 ± 3.87
pKi	9.22±10.15	9.40 ± 10.40	9.70 ± 10.70	6.22 ± 10.40	9.15 ± 10.15

Concentrations are expressed as means and SEMs of the negative log-transformed values (M). BuTAC data is reprinted from Watt et al. 2013 [10].

Xanomeline displayed partial agonist activity at the M_1_ receptor (pEC_50_ = 8.11±8.41 M; %E_max_ = 68.3±5.3%), the M_2_ receptor (pEC_50_ = 7.94±8.87 M; %E_max_ = 55.2±5.1%) and the M_4_ receptor (pEC_50_ = 7.61±8.47 M; %E_max_ = 41.8±2.0%). Xanomeline displayed concomitant partial antagonist activity at the M_1_ receptor (pIC_50_ = 8.83±9.18 M; %inhibition = 15.2±0.47%), the M_2_ receptor (pIC50 = 8.78±8.94 M; %inhibition = 19.4±0.77%) and the M_4_ receptor (pIC_50_ = 6.07±6.62 M; % inhibition = 51.4±2.6%).

Xanomeline demonstrated nearly full antagonist activity at the M_3_ and M_5_ receptors (pIC_50_ = 6.08±6.77 and 5.48±6.14 nM, respectively; and %inhibition = 87.6±6.8 and 81.8±18.5%, respectively).

PTAC also displayed partial agonist activity at the M_1_ receptor (pEC_50_ = 9.43±9.82 M; %E_max_ = 38.1±9.94%), the M_2_ receptor (pEC_50_ = 9.30±11.1 M; %E_max_ = 68.5±5.23%) and the M_4_ receptor (pEC_50_ = 8.83±10.3 M; %E_max_ = 19.4±0.52%), where the receptor agonism of PTAC was weaker both in affinity and efficacy at the M_4_ receptor compared to the M_1_ and M_2_ receptor. PTAC displayed concomitant partial antagonist activity at the M_1_ receptor (pIC_50_ = 9.03±9.38 M; %inhibition = 54.3±1.70%) and the M_2_ receptor (pIC_50_ = 8.46±8.55 M; %inhibition = 16.8±5.49%) and nearly full antagonist properties at the M_4_ receptor (pIC_50_ = 7.61±8.32 M; % inhibition = 92.7±7.49%).

PTAC demonstrated—in accordance with the effect of BuTAC—nearly full antagonist activity at the M_3_ and M_5_ receptors (pIC_50_ = 7.69±8.44 and 6.97±7.33 nM, respectively; %inhibition = 90.45±3.52 and 87.3±7.07%, respectively).

### BuTAC and d-Amphetamine in Antipsychotic-Naïve Monkeys

**Fig 2:**
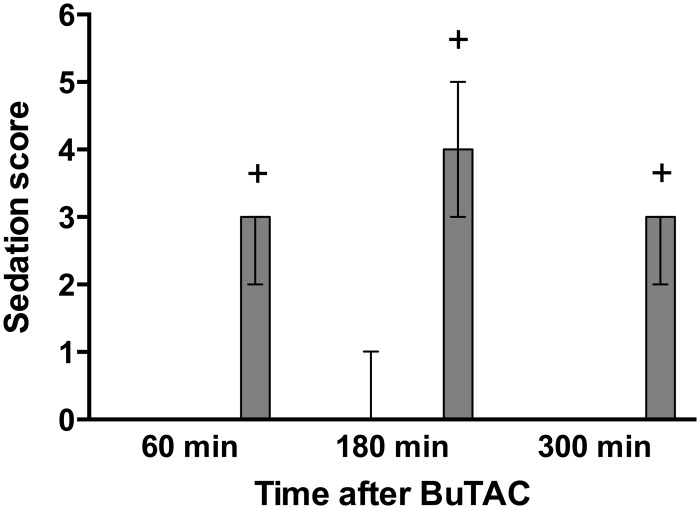
Effect of BuTAC on amphetamine-induced unrest. The behaviour was rated on a scale ranging from 0–6. Data are shown as medians +/- quartiles. +p<0.05 relative to vehicle. *p<0.05 relative to amphetamine, (n = 7).

**Fig 3:**
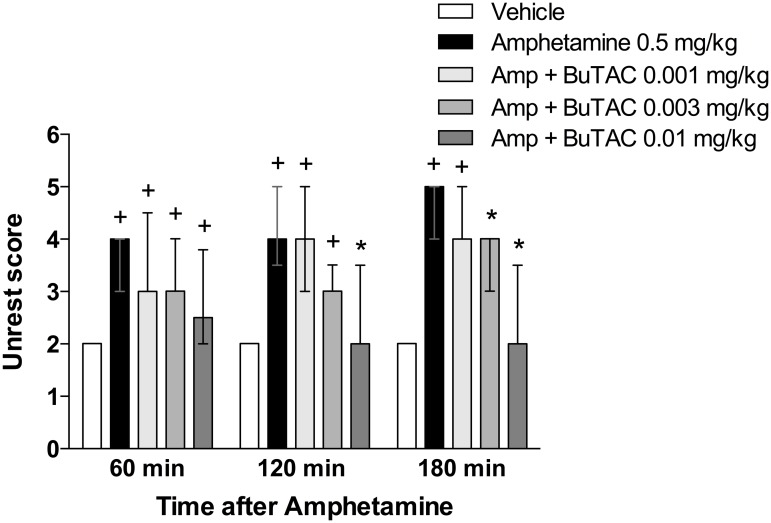
Effect of BuTAC on amphetamine-induced stereotypies. The behaviour was rated on a scale ranging from 0–6. Data are shown as medians +/- quartiles. +p<0.05 relative to vehicle. *p<0.05 relative to amphetamine, (n = 7).

**Fig 4:**
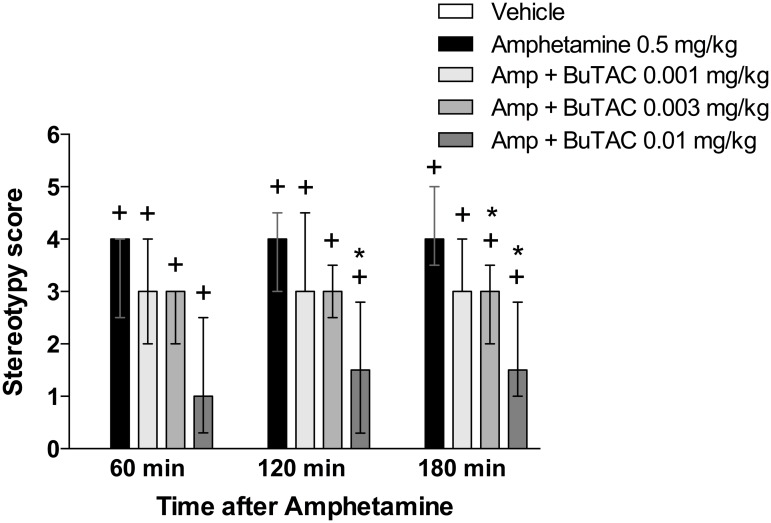
Effect of BuTAC on d-amphetamine-induced arousal. The behaviour was rated on a scale ranging from 0–6. Data are shown as medians +/- quartiles. +p<0.05 relative to vehicle. *p<0.05 relative to amphetamine, (n = 7).

d-Amphetamine at a dose of 0.5 mg/kg significantly increased unrest at all time points (p’s<0.05) (Fig 2). This effect was significantly counteracted by 0.01 mg/kg BuTAC at points 120 and 180 min and 0.003 mg/kg BuTAC at 180 min (Fig 2). Administration of 0.5 mg/kg amphetamine significantly induced stereotypies at all three test times (p’s<0.05 for all) (Fig 3). This behaviour was significantly reduced by BuTAC 0.003 mg/kg at 180 min and by BuTAC 0.01 mg/kg at 120 and 180 min (Fig 3). d-Amphetamine-induced arousal (p<0.05 at all test times) was counteracted by BuTAC 0.001 mg/kg at 60 min and by BuTAC 0.003 and 0.01 mg/kg at 60, 120 and 180 min (p’s<0.05, Fig 4).

Sedation, oral dyskinesia, bradykinesia or dystonia were not observed in monkeys that were given BuTAC and d-amphetamine. Four monkeys vomited when d-amphetamine was combined with 0.003 mg/kg of BuTAC. Six monkeys vomited when d-amphetamine was combined with 0.01 mg/kg of BuTAC. Salivation was observed in four monkeys at 0.001 mg/kg, in five monkeys at 0.003 mg/kg and in four monkeys at 0.01 mg/kg of BuTAC when combined with d-amphetamine. Six monkeys displayed a certain muscular relaxation at BUTAC doses of 0.003 and 0.01 mg/kg combined with d-amphetamine. The monkeys were less active, when this muscular relaxation was observed, but they were awake and attentive. From time to time they got up and walked around, sometimes a bit slower than normal, but they did not display brady-kinesia. We have not observed this kind of “relaxation” when dopamine receptor antagonists were tested in the model.

### BuTAC in antipsychotic-naïve monkeys

**Fig 5:**
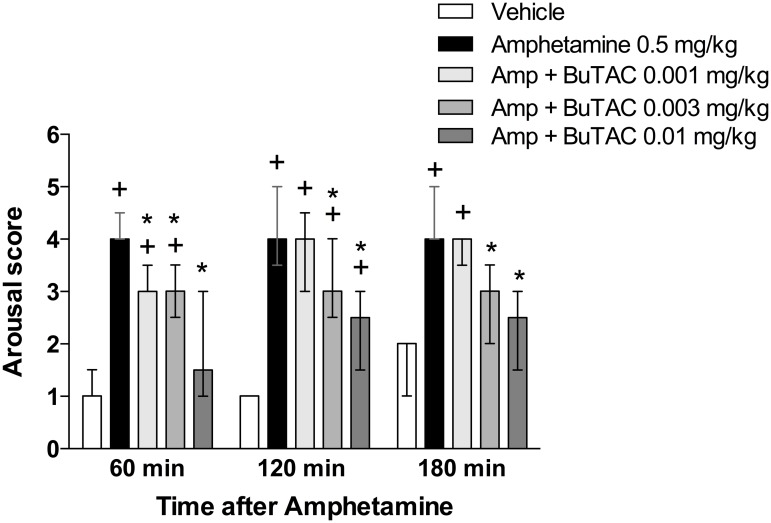
Sedation and unrest after an injection of BuTAC. Sedation and unrest were rated on a scale ranging from 0–6. Data are shown as medians +/- quartiles. +p<0.05 relative to vehicle, (n = 7).

BuTAC alone (0.001, 0.003, 0.01 mg/kg) produced sedation only at the 0.01 mg/kg dose and this was observed at all three test times (p’s<0.05) (Fig 5). There was a concomitant decrease in unrest (p<0.05 after 180 and 300 min) at this dose. Administration of BuTAC produced no significant effect on arousal. Stereotypies, oral dyskinesia, bradykinesia or dystonia were not observed. At the 0.01mg/kg dose of BuTAC all monkeys vomited at least once during the test session (Table 3). At the two lower doses no vomiting was observed. The antiemetic compound domperidone (0.05 and 0.1 mg/kg) dose dependently inhibited BuTAC (0.01 mg/kg)-induced emesis: Seven out of 7 (7/7) monkeys had emesis when BuTAC was administered alone; 3/7 when BuTAC was co-administered with domperidone (0.05 mg/kg) and 1/7 when BuTAC was administered together with 0.10 mg/kg of domperidone (Data provided in S1 Table). Salivation was observed in two monkeys at 0.01 mg/kg of BuTAC (Table 3).

**Table 3:** Overview of BuTAC related side effects.

Treatment	Salivation	Emetic events (vomiting)	Sedation 2 ≤ X ≤ 5
d-amphetamine 0.5 mg/kg (amp)	0	0	0
Vehicle (veh)	0	0	0
BuTAC 0.001 mg/kg / veh	0	0	0
BuTAC 0.001 mg/kg / amp	4 in 7	0	0
BuTAC 0.003 mg/kg / veh	0	0	2 in 6
BuTAC 0.003 mg/kg / amp	5 in 7	4 in 7	0
BuTAC 0.01 mg/kg / veh	2 in 7	7 in 7	7 in 7
BuTAC 0.01 mg/kg / amp	4 in 7	6 in 7	0

The muscular relaxation described in the previous section was observed in all monkeys at 0.01 mg/kg BuTAC and in one monkey at 0.001 and 0.003 mg/kg.

### BuTAC in EPS-sensitized monkeys

Sedation was present in all four EPS sensitized monkeys at a dose of BuTAC of 0.01 mg/kg. Two monkeys displayed mild bradykinesia, i.e. slow movements. Oral dyskinesia and dystonia were not observed. At 0.003 mg/kg no EPS were observed.

## Discussion

Since the historical muscarinic receptor profile of BuTAC has been slightly revised when tested in novel functional in vitro assays using the antibody capture method for G-protein activation [10], we found it relevant to test the muscarinic reference compounds xanomeline and PTAC in the same functional in vitro assays. BuTAC, PTAC and xanomeline were initially developed from the same basic molecular scaffold in an attempt to create compounds with novel muscarinic agonist profiles for novel antipsychotic agents. PTAC and xanomeline have earlier been studied extensively in rodent models predictive of antipsychotic effects and xanomeline has earlier shown efficacy in our d-amphetamine-monkey disease model.

Both the PTAC and the xanomeline profiles were slightly revised compared to the historical data. We have earlier reported that PTAC exhibits partial agonist mode of action at M_2_ and M_4_ receptors and antagonist mode of action at M_1_, M_3_ and M_5_ receptors [15]. The present data shows that PTAC in our novel functional in vitro assays exhibits partial agonist mode of action at M_1_ and M_2_ receptor subtypes and weaker agonist mode of action at M_4_ receptors and full antagonist activity at the M_3_ and M_5_ receptor. We have earlier suggested that muscarinic receptor partial agonists (possibly M_2_ or M_4_) may serve as a new approach to the treatment of schizophrenia [7]. The present in vitro data on PTAC are still in accordance with this suggestion. However, in order to further elucidate the contribution of the different muscarinic subtypes to the potential antipsychotic effect of PTAC, further investigations in mouse genetic deletion models, e.g. M_2_, M_4_ and M_2_/M_4_ knock-out mice, would be valuable.

Quite similar to the profile of BuTAC (full agonist activity at the M_2_ receptor, partial agonist activity at the M_1_- and M_4_ receptor and full antagonist activity at the M_3_- and M_5_ receptor—see Table 3), xanomeline exhibited partial agonist activity at the M_1_- M_2_- and M_4_ receptor and near full antagonist activity at the M_3_- and M_5_ receptor. In line with the partial agonist profile, xanomeline also behaved like an antagonist at the M_1_, M_2_, M_3_, M_4_ and M_5_ receptor subtypes. In concordance with these results, it has earlier been shown by use of muscarinic knock out mice that xanomeline can restore scopolamine-induced prepulse inhibition of startle (PPI) mainly through M_4_ receptor stimulation [16] and that antidopaminergic effects of xanomeline is dependent on M_4_ receptor stimulation [17]. In addition, allosteric enhancers of M_4_ receptor function, e.g. VU0152100 exhibit antidopaminergic effects in mice [18].

In the present study, d-amphetamine-induced behavioural unrest, stereotypies and arousal in antipsychotic naive *Cebus Apella* monkeys, were antagonized by BuTAC, indicating a potential antipsychotic effect of this muscarinic compound.

BuTAC did not induce EPS in EPS naive monkeys. However, when BuTAC, 0.01 mg/kg was tested in the four EPS sensitized monkeys, two monkeys displayed mild bradykinesia, i.e. slow movements, but no signs of oral dyskinesia, dystonia or other EPS commonly found with dopamine antagonist antipsychotics. At lower doses, i.e. 0.001- or 0.003 mg/kg of BuTAC, no signs of EPS were observed. In comparison, the classical antipsychotic compound haloperidol produced dystonia in 4 out of 7 monkeys at a dose of 0.015 mg/kg, which was also the minimal effective dose to inhibit amphetamine-induced behaviour [13].

The special form of muscle relaxation observed in this study was not seen in earlier studies with dopamine receptor antagonists in our laboratory and its mechanism of action is not clarified. The phenomenon is not equivalent to sedation. When our monkeys are sedated, they will to varying degree be drowsy, be yawning, have “heavy eyelids”, curl up their bodies and sometimes sleep. In the present study, sedation was observed when BuTAC was given alone in the two highest doses, i.e. 0.003 and 0.01 mg/kg. When this “relaxation” was observed, the monkeys were less active than normal, sitting motionless for long time periods, sometimes also curled up, but they would be awake and attentive and would react to each other and to external disturbances. From time to time they would get up and move around in the cage, sometimes a bit slower than normal, but without signs of bradykinesia. A parallel but much less potent phenomenon was observed when the muscarinic M_1_/M_4_ partial receptor agonist xanomeline was tested in an earlier study in our laboratory [4].

Sedation was observed at the highest doses of BuTAC (0.01 mg/kg) when administered alone, but was not seen when BuTAC was administered together with d-amphetamine indicating that sedation could not explain the inhibitory effects of BuTAC on d-amphetamine-induced behaviour.

Emetic events were only observed when the highest dose of BuTAC (0.01 mg/kg) was administered together with vehicle. At this dose level 7 out of 7 monkeys had emesis. However, emesis was also observed at a lower dose of BuTAC (0.003 mg/kg) when administered in combination with d-amphetamine, which may be due to the well-known pro-emetic effects of dopamine receptor agonists. Gastrointestinal side effects including emetic events, i.e. vomiting, have been reported in the clinical trial investigating the effect of the muscarinic receptor agonist xanomeline in Alzheimer’s Disease patients [5]. In the clinic, anti-emetic compounds, e.g. the peripheral dopamine receptor blocker domperidone, is widely used to control vomiting [19], and we found that domperidone dose dependently inhibited BuTAC (0.01 mg/kg)-induced emesis (S1 Table). The specific mechanism underlying the antiemetic effect of domperidone on these muscarinic compounds has not been elucidated.

We have recently showed that conditional genetic deletion of muscarinic M_4_ receptors on dopamine D_1_ expressing neurons in the striatum caused a loss of muscarinic agonist-induced reversal of d-amphetamine-induced hyperactivity in mice [17]. In a recent publication, we reported that BuTAC exhibits an atypical antipsychotic profile in the mouse conditioned avoidance response (CAR) *in vivo* test, a rodent model predicting antipsychotic efficacy, by decreasing the avoidance response at doses that do not induce escape failures [10]. When BuTAC was tested in M_2_^-/-^, M_4_^-/-^, and in M_2_/M_4_^-/-^ double-knockout mice the effects of BuTAC in CAR were almost completely absent in M_2_/M_4_^-/-^ mice and the potency of BuTAC was right-shifted in M_4_^-/-^, compared to wild-type and M_2_^-/-^ mice. The data by Watt et al. [10], points to a central role of the muscarinic M_4_ receptor in the effect of BuTAC in CAR. We have earlier shown that xanomeline, traditionally reported to be a muscarinic M_1_/M_4_ preferring partial receptor agonist, antagonised d-amphetamine-induced behaviours in *Cebus Apella* monkeys [4]. The very similar behavioural effects of BuTAC and xanomeline on d-amphetamine-induced behaviour in monkeys also support the involvement of M_4_ receptors in mediating the functional dopamine antagonism in accordance with the antidopaminergic effects of positive allosteric modulators of M_4_ receptors [20,21].

In conclusion, the muscarinic receptor ligand BuTAC (full agonist activity at the M_2_ receptor, partial agonist activity at the M_1_—and M_4_ receptors and full antagonist activity at the M_3—_and M_5_ receptors) exhibits antipsychotic-like behaviour in primates. The result is quite similar to the earlier reported effects of xanomeline (partial agonist activity at the M_1_- M_2_- and M_4_ receptor and near full antagonist activity at the M_3_- and M_5_ receptor) in *Cebus Apella* monkeys [4]. The novel and revised biochemical profiles of the muscarinic ligands BuTAC [10] (PTAC (this paper) and xanomeline (this paper) also indicate that a contribution from the M_1_ and M_2_ muscarinic receptor subtypes may play a role in the antipsychotic-like in vivo effects of these compounds. The present data further substantiate the rationale for the use of muscarinic M_1_/M_2_/M_4_-preferring receptor agonists as novel pharmacological tools in the treatment of schizophrenia.

## Supporting Information

S1 Table Emesis BuTAC / Domperidone. (PDF)
