# Supplementary material for: Antipsychotic-Like Effect of the Muscarinic Acetylcholine Receptor Agonist BuTAC in Non-Human Primates
Source: PLoS One. 2015 Apr 16;10(4):e0122722. doi: 10.1371/journal.pone.0122722 (PMC4399838; doi:10.1371/journal.pone.0122722)
Supplement: S1 Table — (PDF) [file pone.0122722.s001.pdf]

**S1 Table. Emesis BuTAC / Domperidone**

| Treatment | Emetic events (vomiting) |
|---|---|
| Vehicle | 0 in 7 |
| BuTAC 0.01mg/kg | 7 in 7 |
| BuTAC 0.01mg/kg / domperidone 0.05mg/kg | 3 in 7 |
| BuTAC 0.01 mg/kg / domperidone 0.10 mg/kg | 1 in 7 |

Domperidone was purchased from Tocris Bioscience (Bristol, United Kingdom) and dissolved in 0.9% saline. The compound was administered subcutaneously where after the animals were observed for the following 300 minutes. Numbers of monkeys exhibiting emetic events were measured.
